# Adenoviral Inciting Antigen and Somatic Hypermutation in VITT

**DOI:** 10.1056/NEJMoa2514824

**Published:** 2026-02-12

**Authors:** Jing Jing Wang, Linda Schönborn, Theodore E. Warkentin, Luisa Müller, Thomas Thiele, Lena Ulm, Uwe Völker, Sabine Ameling, Sören Franzenburg, Lars Kaderali, Ana Tzvetkova, Alex Colella, Tim Chataway, Chee Wee Tan, Bridie Armour, Alexander Troelnikov, Lucy Rutten, James McCluskey, Roland Zahn, Tom P. Gordon, Andreas Greinacher

**Affiliations:** 1 College of Medicine and Public Health, Flinders Health and Medical Research Institute, Flinders University, Adelaide, South Australia (SA), Australia; 2 Department of Immunology, South Australia (SA) Pathology, Adelaide, SA, Australia; 3 Department of Transfusion Medicine, Universitätsmedizin Greifswald, Greifswald, Germany; 4 Department of Pathology and Molecular Medicine, McMaster University, Hamilton, ON, Canada; 5 Friedrich Loeffler-Institute of Medical Microbiology, Universitätsmedizin Greifswald, Greifswald, Germany; 6 Department of Functional Genomics, Universitätsmedizin Greifswald, Greifswald, Germany; 7 Institute of Clinical Molecular Biology, Christian-Albrechts-University of Kiel, Kiel Germany; 8 Institute of Bioinformatics, Universitätsmedizin Greifswald, Greifswald, Germany; 9 Department of Medicine, Faculty of Health and Medical Sciences, University of Adelaide, Adelaide, SA, Australia; 10 Johnson and Johnson, Innovative Medicine, Leiden, The Netherlands; 11 Department of Microbiology and Immunology, The University of Melbourne, Melbourne, VIC, Australia

## Abstract

**BACKGROUND:**

Vaccine- (or virus-) induced immune thrombocytopenia and thrombosis (VITT) is a rare prothrombotic complication following adenoviral vector-based Covid-19 vaccination or natural adenovirus infection. VITT is mediated by platelet-activating antibodies against the highly cationic protein platelet factor 4 (PF4). The underlying antigen trigger and immunopathogenesis remain unknown.

**METHODS:**

We determined the amino acid sequences of anti-PF4 antibodies from 21 patients with VITT using antibody proteomics and sequenced immunoglobulin light-chain hypervariable genes from 100 VITT patients. To identify an adenoviral trigger, we utilized the antigen binding fingerprints of anti-PF4 and anti-adenovirus protein antibodies to identify a shared serum clonotype, subsequently mapping the mimicking linear epitope using adenovirus protein peptides and recombinant anti-PF4 VITT antibodies.

**RESULTS:**

Genomic and proteomic profiling of VITT antibodies revealed a shared immunoglobulin light-chain allele 3-21 (IGLV3-21*02 or *03) containing a critical somatic hypermutation, K31E (positively charged lysine to negatively charged glutamic acid; position-31). Only antibodies purified against adenoviral core protein VII (pVII) contained anti-PF4 species matching the VITT fingerprint; antibodies against intact virions or other adenoviral proteins did not. Cross-reactive IgGs were mapped to a basic linear epitope on pVII. Notably, a pathogenic anti-PF4 VITT antibody, back-mutated to germline (K31), lost its prothrombotic activity in-vitro and in-vivo and preferentially bound pVII, directly supporting the role of the hypermutation in antigenic shift from adenovirus pVII to PF4.

**CONCLUSIONS:**

VITT occurs when individuals harboring immunoglobulin light-chain allele 3-21*02 or *03 develop a specific somatic hypermutation within antibodies recognizing a restricted adenoviral core protein pVII epitope, which misdirects antibody targeting towards PF4. (The study was approved by the ethics board of the University Medicine Greifswald (BB 052/21a) and registered at EU PAS Register (EUPAS45098, full study protocol available under https://www.encepp.eu/encepp/viewResource.htm?id=47159) and the German Clinical Trials Register (DRKS00025738).)

(Funded by Deutsche Forschungsgemeinschaft and others; Trial Registration DRKS00025738.)

## INTRODUCTION

Anti-platelet factor 4 (PF4) antibodies cause the severe adverse reaction, vaccine-induced immune thrombocytopenia and thrombosis (VITT).^1–3^ VITT is specific for adenovirus vector-based Covid-19 vaccines, ChAdOx1 nCoV-19-S [AstraZeneca] and Ad26.COV2.S [Johnson & Johnson/Janssen].^4^ The common feature of these two vaccines is the adenovirus particle,^5^ albeit derived from distinct adenovirus serotypes: ChAdOx1 is a chimpanzee adenovirus, and Ad26 a human adenovirus. Remarkably, VITT also occurs rarely after natural adenovirus infection,^6–8^ suggesting that a highly conserved structure within different adenovirus subtypes potentially induces the anti-PF4 response. This notion is corroborated by the uniform structure of the anti-PF4 antibodies induced in the VITT immune response. The anti-PF4 antibodies isolated from sera obtained from patients with VITT after vaccination or natural adenovirus infection^9^ are typically monoclonal or oligoclonal.^10,11^ They exhibit light and heavy chains with nearly identical (“stereotyped”) molecular fingerprints among patients. This allowed us to generate recombinant versions of these antibodies, enabling detailed *in-vitro* and *in-vivo* studies.^12,13^

Given that high-titer anti-PF4 antibodies appear as early as 5 days post-vaccination, they likely form as part of a secondary (boosted) immune response. Considering the virtually identical fingerprints between the anti-PF4 antibodies after vaccination and adenovirus infection, we hypothesized that anti-PF4 antibodies likely emerge from a memory anti-adenoviral response. Here we pinpoint the antigen inducing VITT as a cross-reactive determinant shared between PF4 and the highly conserved, highly abundant adenoviral core protein VII (pVII), which binds to the viral genome and mediates its nuclear transport.^14^ Importantly, pVII shares similarities to PF4 in both charge and binding properties to natural ligands like DNA.^14^ Strongly stimulated B cells undergo somatic hypermutation, introducing single base changes into the rearranged variable regions of IgG at a rate of approximately one mutation per kilobase per generation,^15^ driving affinity-based selection.^16^ We demonstrate that a specific genetic background (IGLV3-21*02/*03 allele) together with a specific somatic hypermutation causes a striking shift in antibody specificity from pVII to PF4, resulting in pathogenic “VITT antibodies” capable of forming platelet-activating PF4-IgG immune complexes.

## METHODS

Detailed descriptions of methods are given in the Supplementary Appendix.

**Patients,** who provided written informed consent, as described,^17^ and were reported previously,^10,17–19^ presented with typical VITT or pre-VITT after ChAdOx1 nCoV19-S or Ad26.COV2.S vaccination according to the revised Brighton collaboration case definition^4^ (Table 1), or after adenovirus infection.^9^ All patients had platelet-activating anti-PF4 antibodies detected by anti-PF4/heparin enzyme-linked immunosorbent assay (ELISA), with strong reactivity against PF4 alone in a chemiluminescence assay,^7^ and by the PF4-induced platelet activation assay (PIPA).^2^ Furthermore, baseline repository plasma was available from two individuals who had donated blood several weeks before Covid-19 vaccination and subsequent development of VITT.

### Antibody proteomics by mass spectrometry (MS) sequencing, paratope modeling, and recombinant anti-PF4 antibody production

We affinity-purified antibodies from sera obtained from patients with VITT. We used ChAdOx1 virion-coated magnetic beads to isolate antibodies binding to ChAdOx1 surface antigens. We next purified anti-PF4 and anti-core protein VII (pVII) antibodies from these sera using PF4-coupled and pVII-coupled magnetic beads, and, by the same approach, anti-penton, anti-protein IIIa (pIIIa), anti-protein V (pV), and anti-protein VI (pVI) antibodies (all adenovirus proteins).^10^ We separated the heavy and light chains of these affinity-purified antibodies by SDS-PAGE under reduced conditions, analyzed peptides by tandem mass spectrometry, and determined peptide sequences by *de novo* sequencing and International ImMunoGeneTics database matching using Peaks studio XPro software (Bioinformatics Solution Inc., Waterloo, ON, Canada). Using the amino acid sequences of anti-PF4 antibodies obtained by mass spectrometry, we generated two recombinant anti-PF4 IgG1 antibodies by reverse engineering^12^ (CR22046, CR23004). In both recombinant antibodies, we also engineered back-mutations to the germline by exchanging amino acid glutamic acid at position-31 of the light chain hypervariable region to germline lysine (E31K) (E31K-CR22046 and E31K-CR23004). For CR22046, we also generated a second antibody variant, changing the first aspartic acid at position-50 of the DDSD motif in the light chain complementarity-determining region 2 (LCDR2) encoded by IGLV3-21*02/*03 to tyrosine encoded by the alleles IGLV3-21*01 or *04 (D50Y-CR22046) (Figure S1). Recombinant antibodies were characterized by the same methods as the original sera. Human serum contains proteins that may interfere with PF4-platelet interaction,^20^ so we dissolved them in normal (anti-PF4 antibody-negative) human serum.

### Assessment of the immunoglobulin light chain hypervariable region gene

DNA purified from peripheral EDTA blood was sequenced on an Illumina NovaSeq 6000 S4 Flowcell using 150bp paired-end reads. The genes encoding the hypervariable region of the immunoglobulin light chain were retrieved from the genome.

### Adenovirus and adenovirus domains

Microtiter plates were coated with adenovirus vectors ChAdOx1-S, Ad26.COV2.S, and recombinantly produced adenovirus proteins (GenScript): penton, pIIIa, pV, pVI, and pVII, and a peptide library of the ChAdOx1 pVII protein (Mimotopes, Melbourne, Australia; peptide sequences in Supplementary Figure S2) to test binding of antibodies from VITT sera and the recombinant anti-PF4 antibodies and their back-mutated counterparts.

### Animal experiments

The recombinant antibody CR22046 or its back-mutated variant E31K-CR22046 were given intravenously (1.5μg/g bodyweight/day) for 5 consecutive days to a transgenic mouse line expressing human PF4 and FcγIIa receptors with an additional knockout for mouse PF4 on a C57BL/6J background (B6(Cg);SJL-Pf4tm1 Tg(PF4) Tg(FCGR2A)).^21–23^

## RESULTS

### Characterization of VITT antibodies

**Figure 1.**
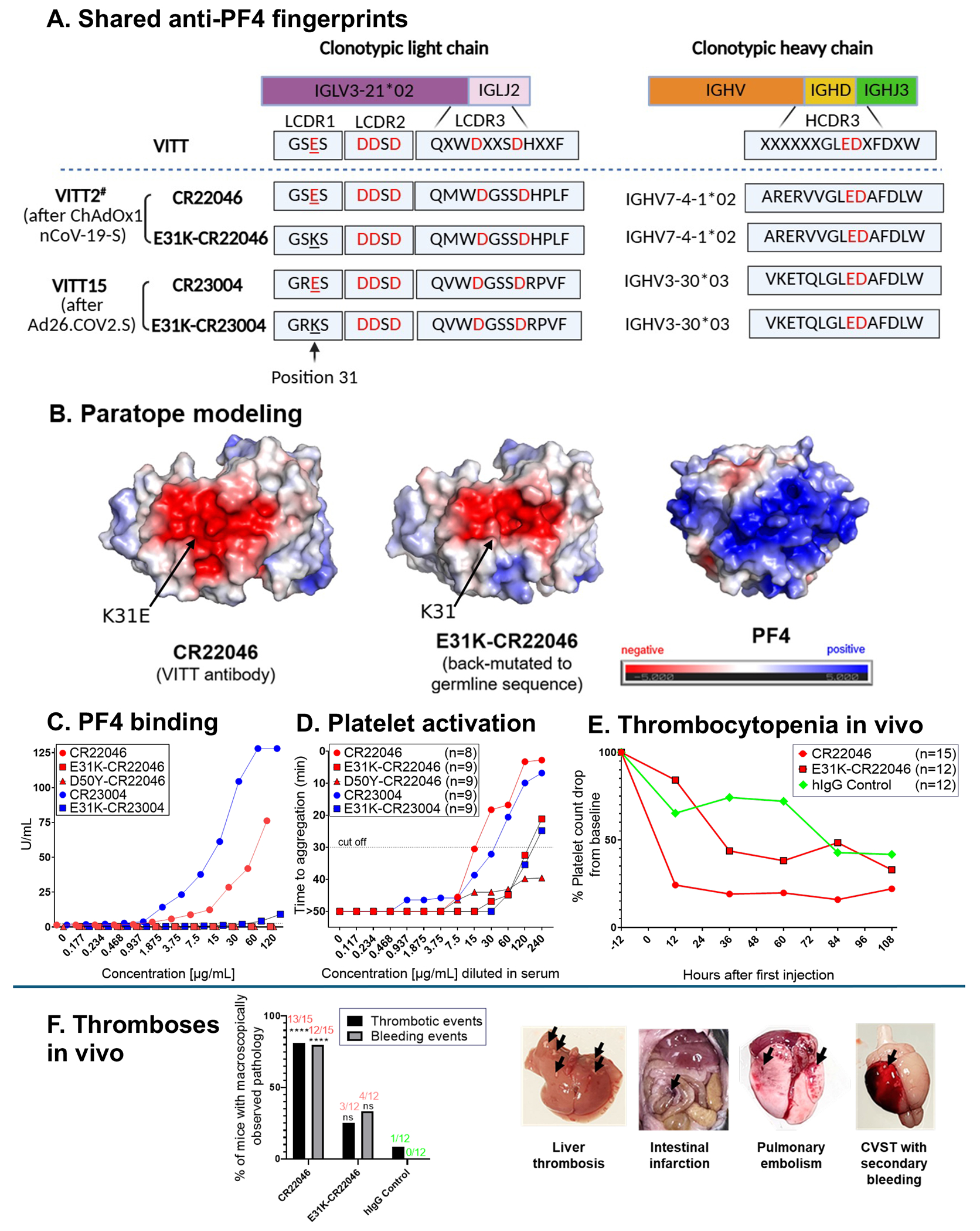
Characterization of anti-PF4 antibodies **Panel A** shows the anti-PF4 fingerprints of the antibodies used in this study. The top of panel A shows consensus anti-PF4 antibody fingerprints from patients with classic VITT, specified by a single immunoglobulin lambda variable 3-21*02 (IGLV3-21*02) light chain paired with a single heavy chain which expresses a shared motif (ED) in the heavy chain third complementarity-determining region (HCDR3). Below the dotted line the recombinant anti-PF4 antibody fingerprints derived from two subjects with VITT after vaccination with ChAdOx1 nCoV-19-S or Ad26.COV2.S as shown. The upper sequence shows the fingerprint of the antibodies as derived from patient serum. Their anti-PF4 signatures are remarkably conserved with acidic residues shown in red (D = aspartic acid; E = glutamic acid). All antibodies express a strongly acidic DDSD motif, a basic lysine (K) to acidic glutamic acid (E) mutation at position-31 (Kabat^35^; K31E; underlined) in the light chain complementarity-determining region 1 (LCDR1), and identical LCDR3 lengths with two equally spaced “D” residues combined with an immunoglobulin lambda joining 2 (IGLJ2). Their heavy chains are of identical HCDR3 lengths (a marker of clonal sharing) with an acidic “ED” motif rearranged with an immunoglobulin heavy joining 3 (IGHJ3). Anti-PF4 antibodies express distinct immunoglobulin heavy variable (IGHV) subfamilies. The lower sequence shows the fingerprint of the recombinant antibodies in which the somatic hypermutation of glutamic acid (E) at position-31 of LCDR1 has been back-mutated to the germline lysine (K) (E31K-CR22046 and E31K-CR23004). Data of VITT2^#^ was previously published as VITT2.^10^ **Panel B** shows representative paratopes modeled by AlphaFold3 webserver. The structures represent the heavy and light chain variable domains of a VITT anti-PF4 antibody induced by adenoviral vector-based Covid-19 vaccination and its variant back-mutated to the germline-sequence (IGLV3-21 with a lysine at position-31). The antibody variable domains and PF4 tetramer (PDB code 1RHP; www.rcsb.org/structure/1RHP) were visualized with PyMoL. The scale bar indicates surface electrostatic potential and corresponds to acidic (red) and basic (blue) amino acid residues. The acidic amino acid residues (in red) from heavy and light chains form the PF4 binding paratopes. The exchange of glutamic acid with the germline-encoded lysine at position-31 (E31K) results in a major alteration of the paratope, disrupting its negative charge by removing an acidic residue predicted to bind to basic region of PF4. **Panel C** shows PF4 binding activity of the recombinant anti-PF4 antibodies derived from the two patients with VITT. CR22046 and CR23004 (red and blue circles, respectively) bound in a concentration-dependent fashion to PF4 (Anti-PF4 antibody chemiluminescence assay, Werfen, Barcelona, Spain) but not to PF4/heparin complexes (supplementary material, Figure S3) in two chemiluminescence assays at concentrations of 0.117-120 μg/mL. Antibody concentrations plotted as log^2^-scale. In contrast, their back-mutated counterparts, E31K-CR22046 and E31K-CR23004 (red and blue squares), in which the glutamic acid at position-31 of the hypervariable region of the light chain was back-mutated to lysine (germline DNA sequence), lost their PF4 binding capacity. This was also observed for the D50Y-CR22046 variant (red triangle), in which we changed the allele sequence from IGLV3-21*02 or IGLV3-21*03 to an IGLV3-21*01 or *04 allele sequence by targeted mutation. Symbols below the cutoff (2.6 U/mL) might partially overlap. **Panel D** shows platelet activation by recombinant anti-PF4 antibodies. When incubated with washed platelets, CR22046 and CR23004 activated platelets dose-dependently, while their back-mutated counterparts E31K-CR22046 and E31K-CR23004, as well the D50Y-CR22046 variant, did not, up to a concentration of 120 μg/mL. The dashed line shows the cutoff of the functional assay. Mean values of n=8 and n=9 different donor platelets are shown to enable readability. Antibody concentrations (x-axis) plotted as log^2^-scale. Individual data points are shown in the supplementary material (Figure S6). **Panel E** shows the decrease in platelet counts (% from baseline; mean) in mice transgenic for human PF4 and FcγIIa receptors, and knockout for mouse PF4 on a C57BL/6J background, receiving 1.5μg/g bodyweight/day for 5 consecutive days of either CR22046, its back-mutated variant E31K-CR22046 or human IgG control. Due to euthanasia in the course of the experiments, not all mice contributed to the later measurement time points. An overview of the exact numbers of mice in each group contributing to the different measurement time points is given in supplementary Figure S7.The delayed decrease in the platelet count in the control animals is caused by frequent blood sampling and also occurs if only saline is given in the experiment (supplementary Figure S7). **Panel F** shows the percentage of mice with macroscopic thrombosis or bleeding and representative images of liver, intestines, lung, and brain. 13/15 mice [86.7%] which received CR22046 developed thrombotic complications (8 mice showed thromboses at multiple sites). Secondary cerebral hemorrhage was caused by cerebral venous sinus thrombosis. In mice which received the back-mutated E31K-CR22046 variant, thrombotic complications occurred in only 3/12 mice (1 mouse showing multiple thrombosis in liver and cerebral venous sinus (CVST), 2 mice with pulmonary embolism).

We determined the amino acid sequences of the heavy and light chains of purified anti-PF4 antibodies from 21 patients with VITT after either ChAdOx1 nCoV-19-S (n=18) or Ad26.COV2.S Covid-19 (n=3) vaccination. All antibodies possessed similar molecular fingerprints with the common “ED” motif in the heavy chain and restricted IGLV3-21*02/*03 light chain, consistent with Ig light chain genotyping (Table 1; Figure 1A). Notably, a striking shared somatic mutation was observed in the light chains across different patients, which resulted in an exchange of a positively charged lysine (K) to a negatively charged glutamic acid (E) mutation at position-31 (K31E) (Table 1; Figure 1A). We confirmed through germline immunoglobulin G sequencing in 18 subjects with available DNA that this E31 was a somatic hypermutation, i.e., not present as a germline polymorphism.

This finding was confirmed by genome sequencing of an additional 82 patients with VITT,^17^ all of whom had the IGLV3-21*02/*03 allele encoding lysine at position-31 (K31) in their germline sequence of the hypervariable light chain region.

This negatively charged glutamic acid at position-31 of the light chain, formed by somatic mutation, together with the negatively charged “ED” binding motif of the VITT antibody heavy chain hypervariable region (Figure 1A), forms a strongly negatively charged paratope, facilitating strong binding to positively charged PF4 (Figure 1B). In contrast, in three healthy individuals developing non-pathogenic anti-PF4 antibodies after ChAdOx1 nCoV19-S vaccination,^24^ the resulting antibodies were low-titer and non-platelet-activating. Furthermore, antibody sequencing revealed evidence of multiple antibody clonotypes lacking both the typical IGLV3-21*02/*03 allele and the K31E mutation.

From the pre-vaccination plasma of two individuals who subsequently developed VITT after ChAdOx1 nCoV19-S vaccination, we isolated non-pathogenic, polyclonal anti-PF4 antibodies. In both individuals we identified the prototypical anti-PF4 light chain encoded by IGLV3-21*02 expressing the germline-encoded lysine at position-31 (K31). In one individual, a rare somatic variant with a K31E mutation was observed, present in 2% of the LCDR1 peptides detected in the proteomic analysis. In contrast, their VITT anti-PF4 antibodies generated in the acute-phase were dominated by the typical K31E mutation (Table 1), absent in the germline DNA of these two individuals.

### Assessment of somatic hypermutation using recombinant anti-PF4 VITT antibodies

We next generated two recombinant VITT IgG antibodies (CR22046 and CR23004^13^) from patients developing VITT after ChAdOx1 nCoV19-S or Ad26.COV2.S, respectively (Figure 1A). Both recombinant antibodies possessed activity profiles identical to typical VITT antibodies,^13^ i.e., they bound PF4 (Figure 1C) but not PF4/heparin complexes (Figure S3), and activated platelets in a PF4-dependent fashion (Figure 1D). Additionally, dosing of CR22046 (n=15) led to a rapid and pronounced platelet count decrease (−79.3%) (Figure 1E) and thromboses in 13/15 hPF4-hFcγRIIa-transgenic mice (86.7%), including cerebral venous sinus and splanchnic vein thromboses (Figure 1F).^23^

To understand the functional role of K31E in VITT pathogenesis, we examined the recombinant antibodies CR22046 and CR23004, back-mutated to express the germline lysine (K) residue at position-31 of the hypervariable region of the light chain (E31K-CR22046 and E31K-CR23004). Both back-mutated recombinant antibodies showed a major reduction of binding to PF4 in the chemiluminescence test and required very high concentrations (≥120 μg/mL) to induce platelet activation in the functional PF4-dependent assay (Figure 1C&1D). *In-vivo*, the back-mutated E31K-CR22046 caused thromboses only in 3/12 mice (Figure 1F) and exhibited the same platelet count profile as the negative control (Figure 1E). We conclude that K31E represents a crucial somatic hypermutation, which is required to produce the highly reactive anti-PF4 antibodies characteristic of VITT.

To determine the impact of the IGLV3-21 light chain allele, we substituted the IGLV3-21*02/*03 allele aspartic acid (D) at position-50 with tyrosine (Y) as encoded by the IGLV3-21*01 or *04 allele (D50Y-CR22046). This resulted in loss of PF4 binding and platelet activation (Figure 1C&1D), compared with CR22046.

### Epitope mimicry between adenovirus core protein pVII and PF4

Following the hypothesis that pathogenic anti-PF4 antibodies arise from a misdirected memory response to a shared adenoviral antigen, we next sought to determine whether VITT-related antibody species are present in the antibody response to the adenoviral vector. First, we did not detect any matched light chain or heavy chain motifs unique to pathogenic anti-PF4 clonotypes in antibodies isolated from intact ChAdOx1 virion particles from VITT serum. Further, neither human anti-PF4 antibodies (purified from VITT sera) nor recombinant anti-PF4 antibodies bound to ChAdOx1 or Ad26 virion coated on ELISA plates (Figure S4). These findings argue against an inciting antigen on the adenoviral surface.

**Figure 2.**
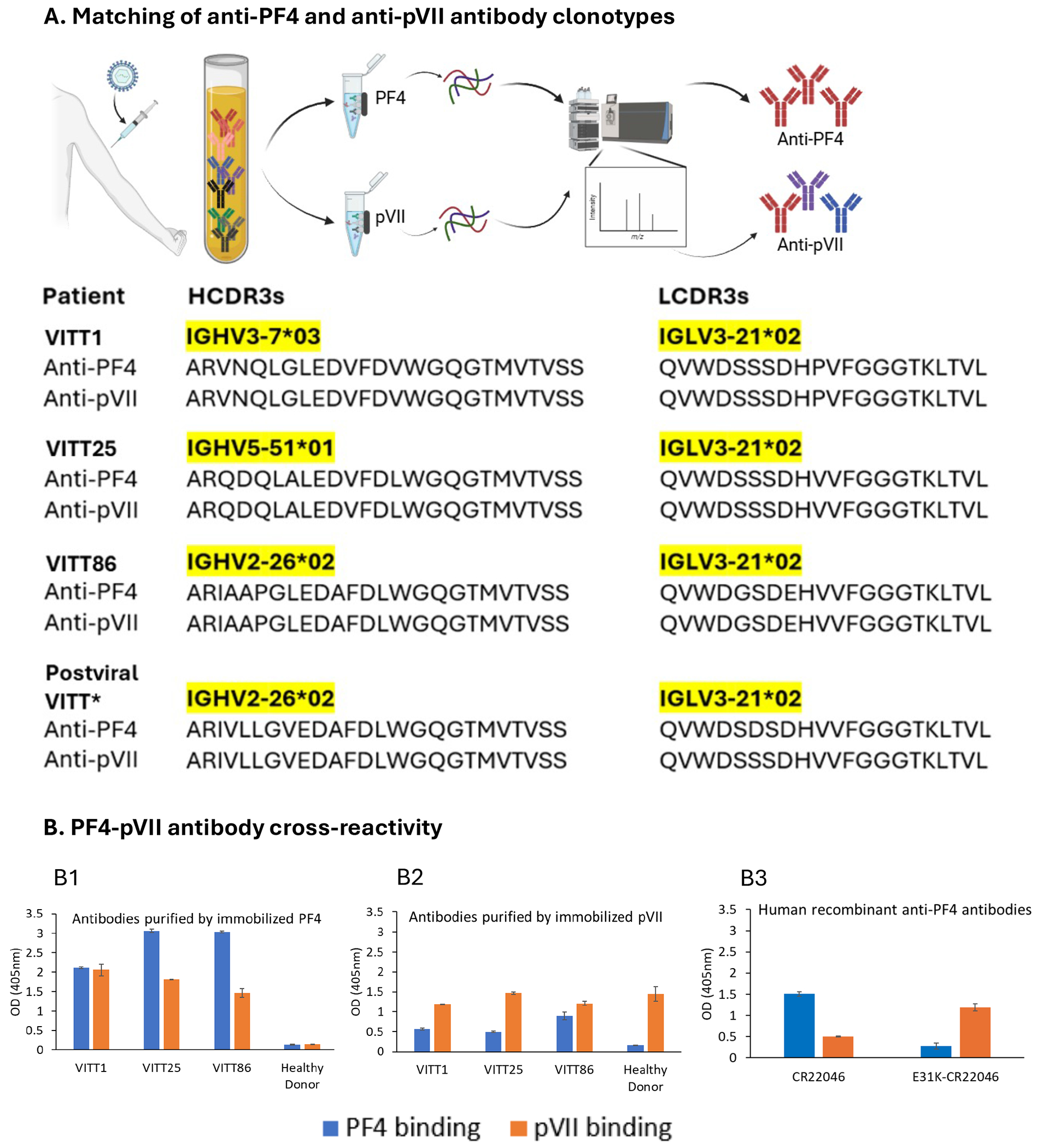
Serum anti-PF4 and anti-pVII clonotype analyses and immune cross-reactivity. **Panel A** shows matching of anti-PF4 and anti-pVII antibody clonotypes. Specific antibodies purified from sera obtained from patients with VITT using PF4- or pVII-coupled Dynabeads are digested with enzymes to generate peptides for liquid chromatography-tandem mass spectrometry. The matched serum anti-PF4 and anti-pVII clonotypes, shown schematically in red, are identified by heavy (H)- and light (L)-chain complementarity determining region 3 (CDR3) clonotypic barcodes. Data of postviral VITT* was previously published as Patient 1.^9^ **Panel B** shows two-way immune cross-reactivity of bead-purified anti-PF4 (B1), anti-pVII IgGs (B2) or human recombinant anti-PF4 antibodies (B3) derived from sera obtained from patients with VITT with pVII and PF4 antigens by ELISA. Serum of a healthy ChAdOx1 nCoV-19-S vaccinee was included as a healthy donor.

Next we examined responses to adenovirus core proteins, focusing on pVII as a candidate, given its high abundance and similarities in charge and polyanion binding properties to PF4.^14^ Immunoglobulin G purified from four VITT sera against recombinant ChAdOx1 pVII protein was found to be oligoclonal but contained a clonotypic species that matched anti-PF4 antibody fingerprints from the same patient with VITT (Figure 2A). Antibodies purified against each protein (pVII and PF4) revealed reciprocal cross-reactivity (Figure 2B1&2). Anti-pVII antibodies detected in a healthy vaccinee were non-cross-reactive with PF4 (Figure 2B2 “healthy donor”). Furthermore, human recombinant anti-PF4 VITT antibodies also bound to pVII (Figure 2B3). Importantly, the recombinant antibody E31K-CR22046, in which we back-mutated the sequence at position-31 to the germline sequence, lost reactivity to PF4 while showing enhanced binding to pVII compared with its K31E counterpart (Figure 2B3). In contrast, none of our controls—the purified anti-penton, anti-pIIIa, anti-pV or anti-pVI antibodies—contained clonotypic species that matched anti-PF4 fingerprints from the same patient with VITT.

**Figure 3.**
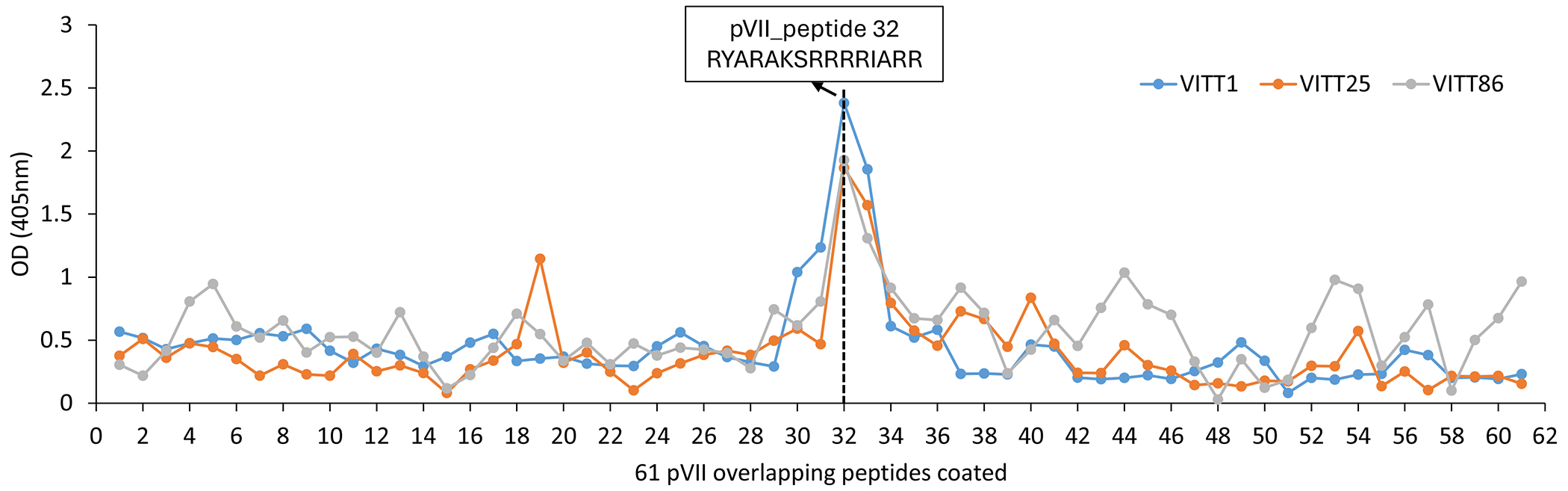

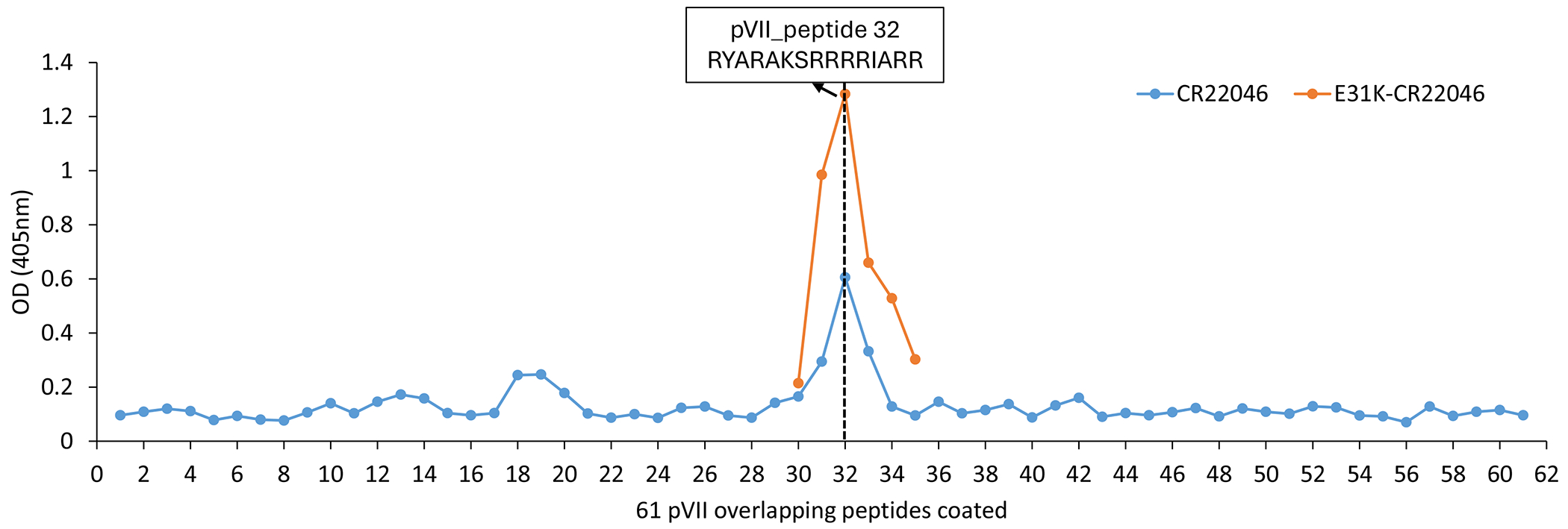

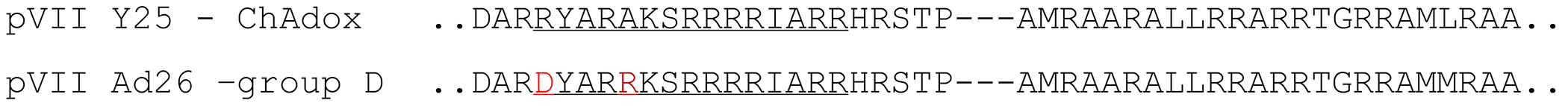

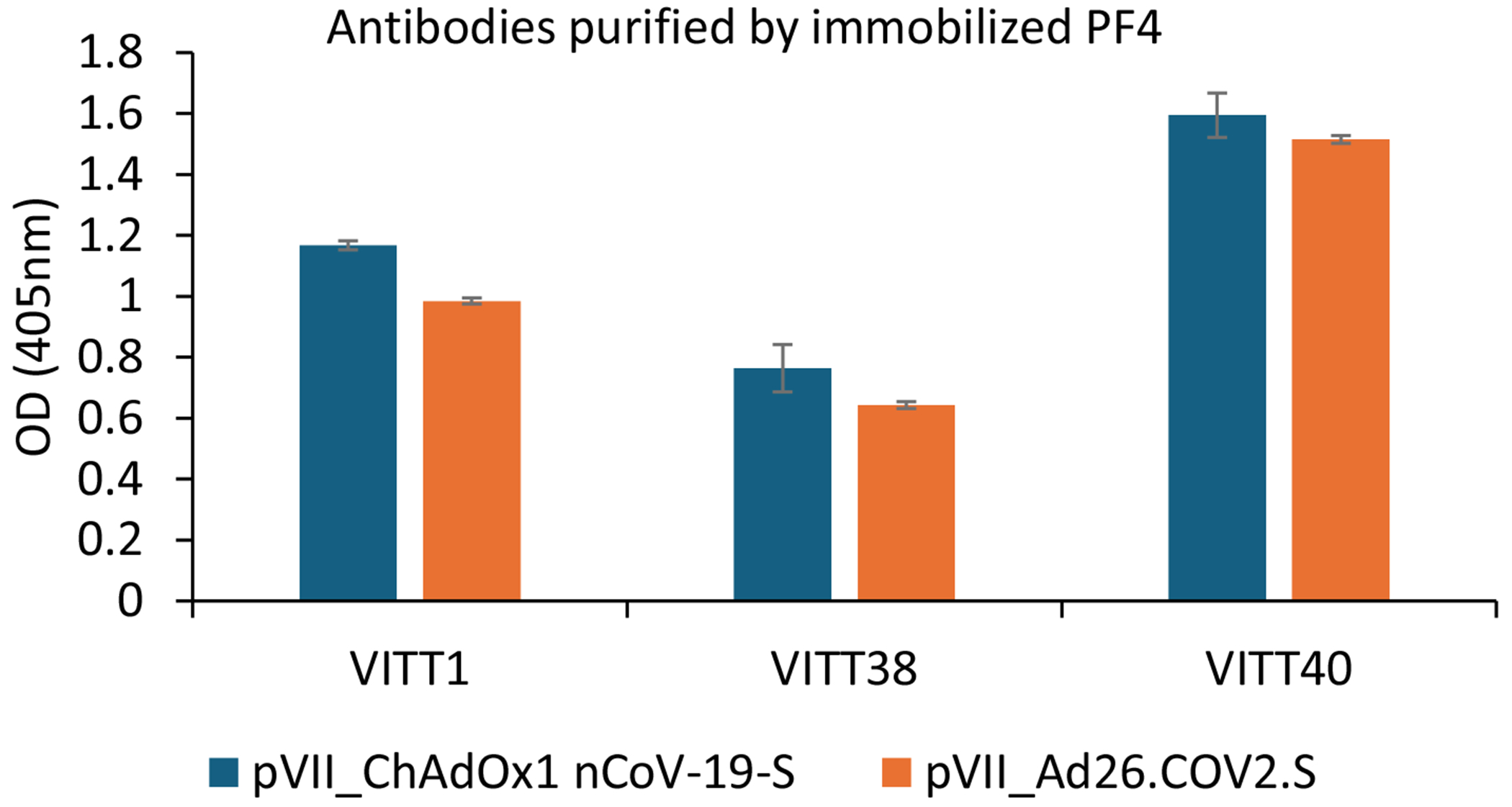
Identification of the pVII epitope **Panel A** A set of 61 15-mer peptides overlapping by 11 amino acids mapping ChAdOx1 pVII were produced and coated to a microtiter plate. The mimicking pVII epitope is identified as a 15-mer linear peptide epitope, indicated within the rectangle, as shown in panel A by binding of anti-PF4 IgGs purified from three VITT sera and in **Panel B** by binding of two human recombinant anti-PF4 antibodies derived from VITT sera (amino acid sequences of these overlapping pVII peptides are shown in Figure S2). **Panel C** shows that the identified pVII epitope (peptide 32, underlined) is highly conserved with nearly identical amino acid sequences in pVII of ChAdOx1 and Ad26. **Panel D** Accordingly, anti-PF4 antibodies purified from VITT sera bind with similar strength to both peptides from different adenovirus species, regardless of whether VITT occurred after vaccination with ChAdOx1 nCoV19-S (VITT1) or Ad26.COV2.S (VITT38 and VITT40). Figure S8 shows binding of the recombinant antibody CR23004 and its counterpart back-mutated to germline E31K-CR23004, against PF4, ChAdOx1 pVII, and the pVII peptides Ad26-pVII **D**YAR**R**KSRRRRIARR and ChAdOx1-pVII **R**YAR**A**KSRRRRIARR.

We next mapped the binding site of the anti-PF4 IgGs purified from VITT sera using overlapping pVII synthetic peptides. Strongest binding occurred to a 15-mer peptide reflecting a single linear epitope in the middle of the ChAdOx1 pVII (Figure 3A). This finding was fully recapitulated by our human recombinant anti-PF4 antibodies. Consistent with all other experiments, the back-mutated anti-PF4 antibody E31K-CR22046 showed increased binding to the 15-mer peptide (Figure 3B). The identified 15-mer peptide (RYARAKSRRRRIARR) is highly conserved in ChAdOx1 and Ad26 (Figure 3C; 3D) and predicted to form an alpha-helix analogous to the PF4 epitope in VITT^25^ (Figure S5).

## DISCUSSION

Molecular mimicry between the adenoviral core protein pVII and PF4, combined with somatic hypermutation transforming an anti-pVII immune response to a misdirected anti-PF4 immune response, is a fundamental pathobiological mechanism of VITT. Immune cross-reactivity between pVII and PF4 results from shared, highly positively-charged epitopes with structural similarity in alpha helixes of both proteins. Nonetheless, production of pathogenic, high-avidity anti-PF4 reactive antibodies requires additional specific genetic features. The first prerequisite is a genetic predisposition harboring the allele of IGLV3-21*02/*03 of the hypervariable region of the immunoglobulin light chain. This is supported by our finding that it was present in 99 of 100 patients with VITT (versus the expected background frequency of 20 to 60%, depending upon ethnicity^26^). The second prerequisite is a somatic hypermutation exchanging a positively charged lysine to a negatively charged glutamic acid at position-31 of the light chain hypervariable region of the anti-pVII antibodies. We confirm this previously described typical fingerprint of the hypervariable immunoglobulin G regions^9,10^ in all 21 patients with VITT investigated.

Both IGLV3-21*02 and *03 alleles encode the identical DDSD motif in the LCDR2, critical for forming the paratope (the part of the antibody that binds to PF4). The single amino acid difference between these alleles (position-17) is not critical for paratope formation. However, when we substituted this sequence with an IGLV3-21*01 or *04 sequence (D50Y-CR22046), anti-PF4 binding capacity was lost (Figures 1C; S5). The prerequisite for alleles IGLV3-21*02 or IGLV3-21*03 may explain the lower frequency of VITT in Asia, as these two haplotypes are found in approximately 60% of Caucasian populations, but only 20% of Asian populations.^26^

Many healthy individuals with the genetic background of IGLV3-21*02/*03 acquire anti-adenovirus antibodies.^27^ However, induction of VITT requires B-cells specific for the pVII RYARAKSRRRRIARR epitope and an additional, specific somatic hypermutation leading to a negatively-charged amino acid at position-31 of the immunoglobulin G light chain hypervariable region. All pathogenic anti-PF4 VITT antibodies reported before,^9,10^ or sequenced in this study, show either glutamic acid or aspartic acid at this position. The germline DNA of all 100 patients with VITT we investigated encoded lysine at this position, confirming that this mutation is somatic. This specific hypermutation likely occurs in few B-cells, explaining why VITT anti-PF4 antibodies are mono- or oligoclonal. Recently, pathogenic anti-PF4 antibodies in heparin-induced thrombocytopenia were also found to be monoclonal,^28^ but the role of antibody genetics in shaping these responses is unknown.

In VITT antibodies, the key mutation at position-31—from a positively charged lysine residue to a negatively charged glutamic or aspartic acid—results in a striking change in antibody avidity towards PF4 and pathogenicity, as shown by the *in-vitro* and *in-vivo* experiments using our recombinant antibodies, with and without the K31E mutation. This high avidity is the prerequisite to cluster PF4, thereby forming pathogenic, platelet-activating immune complexes containing PF4.^29,30^

When we back-mutated IGLV3-21 residue 31 from glutamic acid to lysine, as encoded by the germline DNA (Figure 1A), the antibodies still bound to PF4 but with low binding activity and activated platelets only in the presence of PF4 at very high antibody concentrations (Figures 1C&D). Consistent with our model, the back-mutated E31K-CR22046 showed stronger binding to pVII (Figure 2B3).

The rapid onset of the IgG-mediated VITT immune response (as early as 5 days post-vaccination) strongly indicates that the anti-PF4 immune response is secondary. In two individuals we identified a plasma sample available from a few weeks before they developed VITT. Analysis of the clonotypes of the anti-PF4 antibodies in both patients indicated that the clones with the somatic hypermutations rapidly expanded by boosting after vaccination. The hypermutation likely occurred during boosting, but in one patient also a rare preformed anti-PF4 antibody clone harboring the K31E mutation may have rapidly expanded. In these patients, onset of VITT after vaccination was at day 5 and 7, respectively.

A novel aspect of this work is that we have leveraged mass spectrometry-based proteomics to define molecular mimicry via clonal matching of anti-PF4 and anti-pVII repertoires. This advances the conventional approach of immune cross-reactivity by providing unambiguous evidence of mimicry at a clonal level.

**Figure 4.**
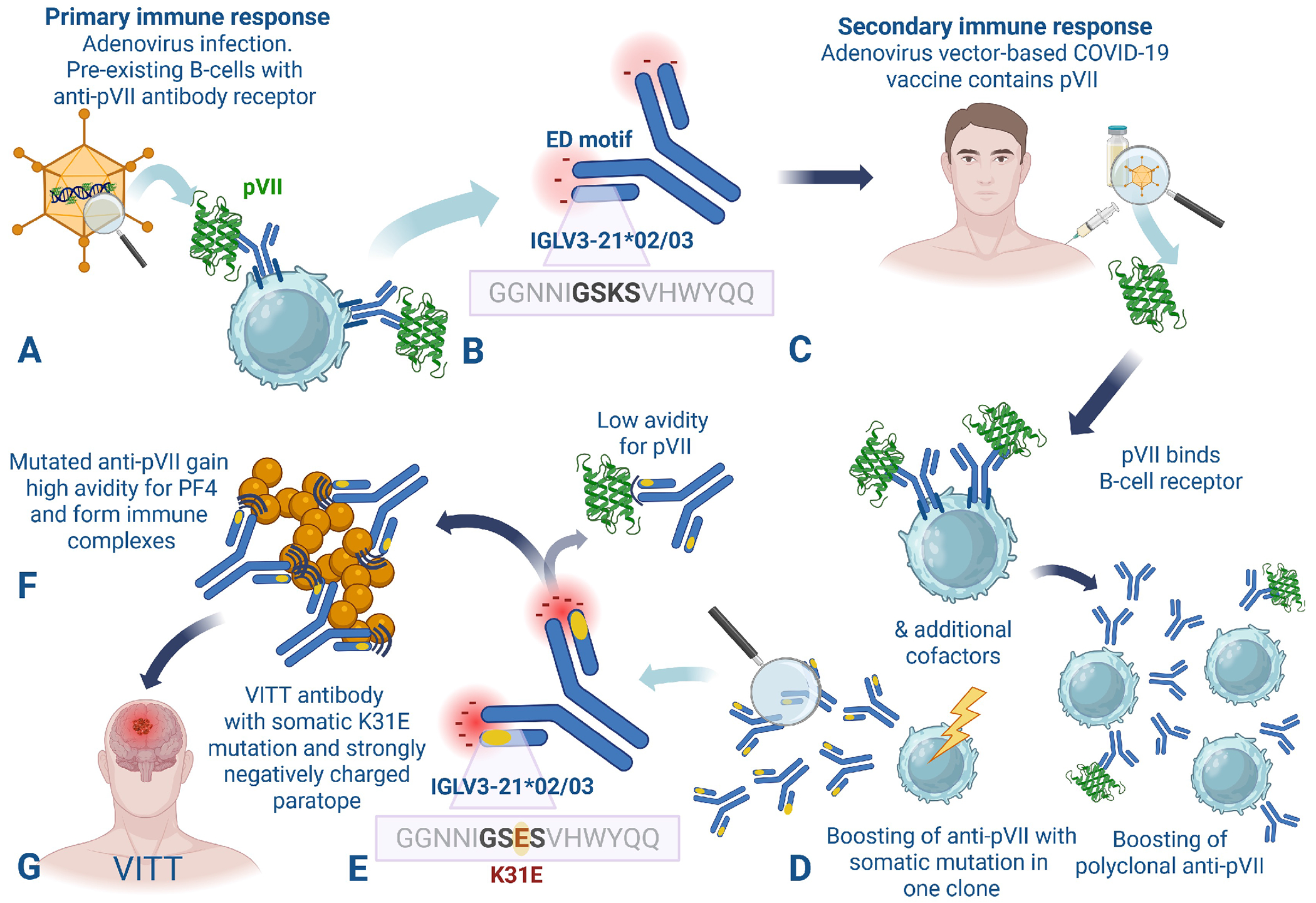
Conceptual model of the immunopathophysiology of vaccine-induced immune thrombocytopenia and thrombosis (VITT). Molecular mimicry between the adenoviral core protein pVII and PF4, combined with a specific somatic hypermutation transforming an anti-pVII immune response to a misdirected anti-PF4 immune response, is a fundamental pathobiological mechanism of VITT. **A)** Primary antigen exposition: A patient with the genetic predisposition of the alleles IGLV3-21*02 or IGLV3-21*03 encoding the hypervariable region of the immunoglobulin light chain comes into contact with numerous viral proteins, including the viral core protein VII (pVII) during adenovirus infection. **B)** The resulting anti-pVII antibodies show the germline sequence of the IGLV3-21*02 or *03 genotype with a positively charged lysine (K) at position-31 and a negatively charged “ED” binding motif of the heavy chain hypervariable region. **C)** The second antigen exposition occurs at the time point of adenovirus vector-based Covid-19 vaccination (or natural adenovirus infection—not shown). The vaccine contains adenoviral proteins including pVII.^5^ pVII binds to the pre-existing B-cell receptor and boosts antibody production. **D)** Boosting causes several B-cell clones to produce polyclonal anti-pVII antibodies. At the same time, rare somatic mutation(s) in one or few B-cell clones occur (alternatively, a preexisting low-level anti-pVII antibody clone harboring the K31E mutation rapidly expands after vaccination; not shown); **E)** The key somatic mutation occurs at position-31— a positively charged lysine (K) is replaced by a negatively charged glutamic acid (E) or aspartic acid (D) — resulting in a highly negatively charged paratope. **F)** This mutation results in antibody reactivity away from pVII and towards PF4. These cross-reacting anti-PF4 antibodies (“VITT antibodies”) now bind with high avidity to PF4, forming large PF4/IgG immune complexes, while binding to pVII remains low-avidity. **G)** These immune complexes induce platelet activation by crosslinking platelet FcγIIa receptors leading to the typical clinical presentation of VITT with thrombocytopenia and thrombosis at unusual sites. This model explains the clinical features of VITT that include the apparent secondary (boosted) immune response with explosive onset of IgG anti-PF4 antibodies as early as 5 days post-vaccination; the very low frequency of VITT (much rarer than HIT) is due to the requirement of a specific somatic mutation in a B-cell specific for a restricted epitope (reflected by the sequence, RYARAKSRRRRIARR) on the background of a specific IGLV haplotype.

Our study has limitations. The critical role of the human specific IGLV3-21*02 or *03 alleles currently precludes mechanistic studies of VITT induction in animal models. This could be overcome by generating human IGLV3-21*02 or *03 transgenic mice. We cannot exclude that additional, not yet identified cofactors (e.g., adenoviral nucleic acids, proteins associated with pVII, spike protein expressed by the adenovirus vector), or other genetic or environmental factors, are required for the pathologic VITT anti-PF4 response (Figure 4). In addition, our study is specific for a misdirected immune response against adenovirus (ChAdOx1 and Ad26) pVII; anti-PF4 disorders induced by other viruses, e.g., cytomegalovirus,^31^ likely have different viral proteins and antibody binding epitopes involved.

The mechanisms underlying pathologic VITT antibodies may help to unravel other poorly understood pathologies, in which a boosted immune response results in pathologic autoantibodies, e.g. post-transfusion purpura in which a boosted anti-human platelet antigen 1a alloantibody response results in formation of autoantibodies destroying the autologous platelets of the patient,^32,33^ and red cell autoantibodies accompanying production of alloantibodies after blood transfusion.^34^

The results of this study are relevant for strategies to make adenovirus vector-based vaccines safer. This vaccination platform has major advantages, e.g., affordability for healthcare systems in low- and middle-income countries and rapid vaccine provision in case of a new pandemic. Identification of molecular mimicry between pVII and PF4 as a fundamental upstream immune mechanism of VITT may now pave the way for developing safer adenoviral vector-based vaccines in which pVII is replaced by non IGLV3-21*02/*03 allele activating analogues.

## Supplementary Material

Supplement

## Figures and Tables

**Table 1:** Clinical characteristics, IGLV3-21 genotyping and mass spectrometric sequencing of serum anti-PF4 light chains from 21 patients with VITT after ChAdOx1 nCoV-19-S or Ad26 COV2.S vaccination.

Characteristics	ID	Phenotype	Anti-PF4/heparin IgG EIA (OD)	PF4-induced platelet activation assay (PIPA)	Genotype	Mass spectrometric sequencing		
						Ig light chain variable region subfamily	LCDR1 K31E ^‡^	LCDR3
**Classic VITT_after ChAdOx1 nCoV-19-S**	**VITT1** ^Ɨ^	CVST with secondary ICH	2.43	strongly positive	N/A	IGLV3-21*02	+	QVWDSSSDHPVFGGGTKLTVL
	**VITT04** ^§^	DVT, thrombosis of internal jugular vein	3.11	strongly positive	*03/*03	IGLV3-21*03	+	QVWDGSSDHPVFGGGTKLTVL
	**VITT05** ^§^	PE, DVT, severe headache	3.41	strongly positive	*03/*01	IGLV3-21*03	+	QVWDGSRDHPVFGGGTKLTVL
	**VITT07** ^§^	CVST with secondary bleeding	3.13	strongly positive	*02/*01	IGLV3-21*02	+	QVWDGSSDHPVFGGGTKLTVL
	**VITT08** ^§^	not provided	2.19	strongly positive	*03/*01	IGLV3-21*03	+	QVWDGSLDHPVFGGGTKLTVL
	**VITT09** ^§^	PVT, headache	2.40	strongly positive	*03/*01	IGLV3-21*03	+	QVWDSSSDHPVFGGGTKLTVL
	**VITT12** ^§^	PE, DVT	1.04	strongly positive	*02/*02	IGLV3-21*02	+	QVWDGSSDHPVFGGGTKLTVL
	**VITT13** ^§^	CVST with secondary bleeding	1.19	strongly positive	*03/*01	IGLV3-21*03	+	QVWDSSSDHVVFGGGTKLTVL
	**VITT16** ^§^	PVT	2.50	strongly positive	*03/*01	IGLV3-21*03	+	QAWDSSSDRPVFGGGTKLTVL
	**VITT19** ^§^	CVST, PE	3.09	strongly positive	*02/*02	IGLV3-21*02	+	QVWDSSSDHPVFGGGTRLTVL
	**VITT25** ^§^	CVST	1.57	strongly positive	N/A	IGLV3-21*02	+	QVWDSSSDHVVFGGGTKLTVL
	**VITT81** * ^§^	DVT, PE, PVT	3.42	strongly positive	*02/*01	IGLV3-21*02	+	QVWDSSSDHPVFGGGTKLTVL
	**VITT86** ^¶^	Arterial stroke, CVST, DVT, PE, PVT	2.80	strongly positive	N/A	IGLV3-21*02	+	QVWDGSDEHVVFGGGTKLTVL
**Classic VITT_after Ad26.COV2.S**	**VITT15** ^§^	DVT, PE, PVT	3.57	strongly positive	*02/*01	IGLV3-21*02	+	QVWDGSSDRPVFGGGTKLTVL
	**VITT38** ^§^	DVT both legs	3.20	strongly positive	*02/*01	IGLV3-21*02	+	QMWDGSSDLPVFGGGTKLTVL
	**VITT40** ^§^	DVT and PE	3.24	strongly positive	*02/*02	IGLV3-21*02	+	QVWHSSRDRVLFGGGTKLTVL
**Classic VITT_preVITT**^#^ **after ChAdOx1 nCoV-19-S**	**VITT06** ^§^	preVITT^#^	3.15	strongly positive	*02/*02	IGLV3-21*02	+	QVWDSSSDHPVFGGGTKLTVL
	**VITT17** ^§^	preVITT^#^	1.69	strongly positive	*02/*02	IGLV3-21*02	+	QVWDGSSDHVLFGGGTKLTVL
	**VITT36** * ^§^	preVITT^#^	3.60	strongly positive	*02/*01	IGLV3-21*02	+	QVWDSGDDHVFFGGGTKLTVL
	**VITT39** ^§^	preVITT^#^	3.26	strongly positive	*02/*01	IGLV3-21*02	+	QVWDGSTDHVLFGGGTKLTVL
	**VITT51** ^§^	preVITT^#^	2.34	strongly positive	*02/*02	IGLV3-21*02	+	QAWESSSDHVVFGGGTKLTVL

All patients had thrombocytopenia (platelet count nadir < 140,000 per mm^3^) even if only thrombosis is listed under “Phenotype”. All patient sera tested strongly positive against PF4 alone in a chemiluminescence assay (data not shown in the Table).

CVST, cerebral venous sinus thrombosis; DVT, deep vein thrombosis; PE, pulmonary embolism; PVT, portal vein thrombosis.

& the IGLV3-21*02 and IGLV3-21*03 alleles differ by one amino acid at position-17 in IGLV3-21 framework region 1, glutamine (Q) for *02 allele and lysine (K) for *03 allele. Both alleles encode the identical DDSD motif in LCDR2. The IGLV3-21*01 allele encode a YDSD motif in LCDR2.

‡ K31E refers to a single amino acid mutation from lysine (K) to glutamic acid (E) at amino acid position-31 in IGLV3-21*02/*03 LCDR1; +, K31E mutation present.

Ɨ Data of VITT1 was previously published in Wang et al. *Blood* 2022 as VITT1^10^

§ Patients VITT04 – VITT81 were patients of the German VITT cohort previously published in Schönborn et al. *J Thromb Hemost* 2023^17^

¶ Patient VITT86 was previously published in Bourguignon et al. *N Engl J Med* 2021^18^

* Individuals with available baseline repository plasma several weeks prior to Covid-19 vaccination and subsequent development of VITT.

# pre-VITT-Syndrome indicates thrombocytopenia and headache but without overt CVST despite brain imaging or other thrombosis, according to Salih et al. N Engl J Med 2021^19^
