# Supplementary material for: Adenoviral Inciting Antigen and Somatic Hypermutation in VITT
Source: N Engl J Med. Author manuscript; Available in PMC 2026 Feb 12. (PMC12900036; doi:10.1056/NEJMoa2514824)
Supplement: Supplement [file NIHMS2126692-supplement-Supplement.pdf]

Table of Contents

Supplementary Materials and Methods 3
Ethical considerations details 3
Anti-PF4/heparin and anti-PF4 IgG enzyme-linked immunosorbent assay (ELISA) 3
Chemiluminescence assays (rapid anti-PF4/heparin and rapid anti-PF4 assay) 3
Heparin- and PF4-dependent platelet activation assay (HIPA and PIPA test) 4
Serum antibody purification 5
Antibody proteomics by mass spectrometry (MS) sequencing 5
Paratope modeling 6
Immune cross-reactivity and pVII epitope mapping by ELISA 6
Recombinant antibody production 6
Genome analysis of 100 patients with VITT 6
Animal Experiments 7
Supplementary Figures 8
Figure S1 8
Figure S2 9
Figure S3 10
Figure S4 11
Figure S5 12
Figure S6 13
Figure S7 14
Figure S8 15
Supplementary References 16

Supplementary Materials and Methods

Ethical considerations details

The study was approved by the ethics board of the University Medicine Greifswald (BB 052/21a) and registered at EU PAS Register (EUPAS45098, full study protocol available under https://www.encepp.eu/encepp/viewResource.htm?id=47159) and the German Clinical Trials Register (DRKS00025738). All animal studies were approved by Landesamt für Landwirtschaft, Lebensmittelsicherheit und Fischerei Mecklenburg-Vorpommern (LALLF-MV; 7221.3-1-009/24).

Anti-PF4/heparin and anti-PF4 IgG enzyme-linked immunosorbent assay (ELISA)

Binding of immunoglobulin G anti-PF4 antibodies obtained from sera of patients with VITT to PF4/heparin complexes or PF4 was measured by a solid phase PF4/heparin ELISA performed in flat-bottomed microwell plates (Thermo Scientific, CovaLink).

PF4/heparin complexes of 0.5 IU/mL unfractionated heparin (UFH; Heparin-sodium, Ratiopharm) and 20 µg/mL PF4 (Chromatec, Greifswald, Germany) were formed in coating buffer (50 mM NaH_2PO_4, 0.1% NaN_3) at RT for 1 h and 100 µL coated per well of the microtiter plate at 4°C. To assess binding of anti-PF4 antibodies, PF4 20µg/mL was coated instead of PF4/heparin complexes. Prior to use, plates were washed five times with washing buffer (150 mM NaCl, 1% Tween20 pH 7.5). 100 µL/microwell of patient serum (1:200 diluted in sample diluent: 0.05 M NaH_2PO_4, 0.15 M NaCl, 7.5% goat normal serum, pH 7.5) was incubated for 60 min at RT, and then washed five times. Horseradish peroxidase-conjugated goat antihuman IgG (Jackson ImmunoResearch Europe Ltd, Ely, UK) was added (1:15,000 dilution in sample diluent). Binding of human IgG was detected by adding chromogenic tetramethylbenzidine substrate (100 µL/microwell; Kementec, Taastrup, Denmark). At 60 min post-incubation, the chromogenic substrate reaction was stopped with 1 M H_2SO_4 (100 µL/microwell) for 10 min at RT, and optical absorbance measured at 450 nm (reference: 620 nm, Tecan, Männedorf, Switzerland) within 10 min. Blank measurements were subtracted from each sample measurement. For quality control each lot of PF4 was tested with a panel of 20 different sera containing anti-PF4/heparin antibodies with known reactivity (1:200 diluted in sample diluent). Each lot of PF4/heparin complex coated plates was controlled with the same quality panel of known anti-PF4/heparin samples for sufficient reactivity. Each ELISA test was controlled with a known weak positive control and a known negative control.

Chemiluminescence assays (rapid anti-PF4/heparin and rapid anti-PF4 assay)

We used two rapid assays to detect anti-PF4/heparin and anti-PF4 antibodies, respectively, using chemiluminescence technology: the HemosIL® AcuStar HIT-IgG(PF4-H) assay, hereafter called rapid anti-PF4/heparin assay (the assay uses PF4/polyvinyl sulfonate complexes [PVS]); and an anti-PF4 antibody assay prototype for the ACL AcuStar, hereafter called rapid anti-PF4 assay.[1] Both assays are two-step chemiluminescence immunoassays consisting of magnetic particles coated either with PF4 complexed to PVS or with PF4 alone. The rapid anti-PF4 assay was designed to detect antibodies that only recognize anti-PF4 antibodies (as seen in VITT) with no or minimal cross-reactivity with anti-PF4/heparin antibodies. Serum (1:11) and citrated-plasma (1:10) in sample diluent were used for analysis. For the testing of recombinant antibodies, antibodies had been diluted in the sample diluent (0.117-120

µg/mL) and then treated like a serum sample. After incubation, magnetic separation, and a wash step, beads were incubated with an isoluminol-labeled anti-human IgG antibody. After a final wash, reagents that trigger the luminescence reaction were added, and the emitted light was measured in U/mL. The cutoff for the rapid anti-PF4/heparin assay was calculated using calibrators, as per manufacturer's instructions. The cutoff of the rapid anti-PF4 assay was determined by a Receiver Operating Characteristic (ROC) curve analysis between VITT and control samples.

Heparin- and PF4-dependent platelet activation assay (HIPA and PIPA test)

Platelet preparation

Platelets were purified from ACD-A anticoagulated whole blood obtained from healthy donors who did not take antiplatelet medications or non-steroidal anti-inflammatory drugs (NSAIDs) during the previous 10 days as demonstrated in the video tutorial available at: https://www.youtube.com/watch?v=hFs-_85YJX4

Platelet-rich plasma (PRP) was centrifuged (7 min at 650 g, without brake) and the platelet pellet washed with Tyrode's buffer containing 0.35% BSA (albumin bovine Fraction V, Serva, Germany), 0.1% glucose (B. Braun, Germany), 2.5 U/mL apyrase (Sigma Aldrich, Germany), 1 U/mL hirudin (Canyon Pharmaceuticals, Switzerland), pH 6.3. After a further centrifugation (7 min at 650 g, without brake), the final platelet pellet was resuspended in a bicarbonate-based suspension buffer consisting of 0.137 M NaCl, 0.027 M KCl, 0.012 M$NaHCO_3$, 0.42 mM NaH_2PO_4, 0.35% BSA, 0.1% glucose, 0.212 M MgCl2, 0.196 M $CaCl_2$, pH 7.2 and adjusted to 300,000 platelets/µL.

PIPA test assessment and interpretation

Human serum contains proteins that interfere with PF4 and PF4-platelet interaction, e.g. fibronectin.[2] To compare the recombinant antibodies within the same matrix as the patients' antibodies, for functional testing we dissolved them in normal human serum which tested negative for anti-PF4 antibodies. Heat-inactivated (56 °C, 30 min) patient serum (20 µL) or recombinant antibodies diluted in heat-inactivated normal serum, and washed platelets (75 µL) was incubated in a microtiter plate (Greiner, Austria) with either buffer, 0.2 aFX U/mL low-molecular-weight heparin, reviparin (Abbott, Germany; if reviparin is not available, enoxaparin can be used; HIPA), 100 IU/mL unfractionated heparin (ratiopharm, Germany), or 10 µL PF4 solution (10 µg/mL, final conc., Chromatec, Germany) in the presence and absence of the FcγIIa receptor-blocking antibody, IV.3 (5 µL added to 75 µL platelets, obtained by cell supernatant, cell line ATCCHB-217, Biometec GmbH). To avoid any effect of thrombin, for all reactions (with the exception of the 100 IU/mL heparin reaction well) hirudin (5 U/mL) was added. Volumes were reduced by 50% in some experiments to safe material with no effect of reactivities.

The microtiter plate was incubated (45 min, RT) on a magnetic stirrer (1000 rpm) with two steel spheres (2 mm diameter, SKF, Mercateo). The transparency of the suspension was assessed using an indirect light source every 5 min. A positive result was defined as activation of platelets (lag time ≤30 min) of at least two of three different donors and inhibition at high heparin concentrations (100 IU/mL). Reactivity lag time of >20-30 min was defined as weak, >10-20 min as moderate, and ≤10 min as strong reactivity.

For quality control each platelet preparation was incubated with low concentrations of collagen to secure sufficient platelet reactivity. Each test included a positive control serum containing anti-PF4 antibodies known to cause platelet activation within 15 to 25 minutes.

Serum antibody purification

Sera from patients with VITT were used to affinity purify antibodies specific to PF4, or the adenovirus proteins pVII, penton, pIIIa, pV, pVI, ChAdOx1 or Ad26 virion particles, as described previously.[3] Native PF4 protein was purchased from ChromaTec (Greifswald, Germany), full length recombinant Chimpanzee adenovirus pVII, penton, pIIIa, pV, and pVI proteins were produced by GenScript (Singapore). Whole virion particles were isolated from ChAdOx1 nCoV-19 vaccines by centrifugation at 53,000xg for 2 hours. MyOne Carboxylic Acid Dynabeads (ThermoFisher) were washed twice in 1ml of 15mM MES buffer followed by activation with 100µL of 1-Ethyl-3-(3dimethylaminopropyl) carbodiimide (10mg/ml) and incubation on a rotator for 30 min at room temperature. Following activation, the beads were washed with 15mM MES buffer and coated with PF4, pVII, pIIIa, pV, pVI, penton, or virion particles, respectively, in 15mM MES buffer. Samples were then incubated overnight on a rotator at room temperature. After antigen coating, the beads were washed twice in 0.1% Tween 20 PBS. Diluted serum was then added to the beads and mixed on a rotator for 2 h at room temperature. After incubation, the bound antibodies were eluted with 100mM glycine elution buffer (pH 11), buffer exchanged with PBS by using 10kd spin columns (Amicon Ultra) and stored at -80°C until required.

Antibody proteomics by mass spectrometry (MS) sequencing

Antibody sequencing was performed as described previously.[3,4] Briefly, purified IgGs were digested with Pierce trypsin protease (ThermoFisher Scientific) and chymotrypsin (Promega), separately. Digested peptides were analyzed with a Dionex Ultimate 3000 UPLC coupled with a Thermo Exploris 480 tandem mass spectrometer (Thermo Fisher Scientific, Waltham, Massachusetts, USA). Samples were analyzed using data-dependent acquisition (DDA) utilizing a 3 second cycle time instrument method. Briefly, ms1 scans were performed using an orbitrap resolution of 60,000 and a scan range from 350m/z-1200m/z. A normalized AGC target of 3e6 with a maximum injection time of 100 ms. An intensity threshold of 2e4 and dynamic exclusion time of 50 sec was employed for all data dependent ms2 scans that were acquired at 15,000 resolution, automatic gain control (AGC) target 1e5, 32% normalized collision energy (NCE) in the higher-energy collisional dissociation (HCD) cell, with a maximum injection time of 200 ms. Protein sequence data analyses were performed by combined de novo sequencing and International ImMunoGeneTics (IMGT) database matching using Peaks studio XPro software (Bioinformatics Solution Inc., Waterloo, ON, Canada). Parameters for database searches, data refinement and Ig variable region subfamily assignments were described previously.[5] High-quality de novo peptides were selected based on sequences having an average local confidence score threshold greater than or equal to 75% and inspected manually to ensure correct assignments. A false discovery rate (FDR) threshold of 1.0% was applied at the peptide level to each data set. The Ig variable region subfamily is assigned from the presence of a unique peptide corresponding to the subfamily. The heavy-chain third complementarity-determining region (HCDR3) were identified by de novo sequencing derived from different enzyme digested peptides.

Paratope modeling

The paratope model of antibody heavy and light chain variable domains was made using AlphaFold3. The surface electrostatic potential for each residue was calculated by Adaptive Poisson-Boltzmann Solver (APBS). The antibody variable domains and PF4 (PDB code 1RHP) were visualized in PyMoL.

Immune cross-reactivity and pVII epitope mapping by ELISA

The immune cross-reactivity of purified antibodies against each protein was determined by an in-house ELISA for reactivity against native PF4 and full-length adenovirus pVII. In brief, maxisorp nunc immune plates (Thermofisher) were coated with 100 µl of individual PF4, pVII at 4 µg/ml in PBS buffer overnight at 4°C. Plates were blocked with PBS 1%BSA (Sigma-Aldrich) and then incubated with each individual purified antibody (equivalent to 1:25 dilution of sera) for 2 h at 37 °C. After incubation, alkaline phosphatase conjugated goat anti-human IgG (γ-chain specific) secondary antibodies were added and incubated for 1 h at 37 °C. Phosphatase substrate (Sigma) was added after extension wash with PBS 0.05% Tween 20. Optical density (OD) at 405nm was measured by a plate reader (Spectramax Id5). Blank measurements were subtracted from each sample measurement.

The pVII epitope was identified by ELISA for reactivity against a set of 15mer peptides with overlap of three residues for Chimpanzee adenovirus pVII protein (Mimotopes, Melbourne, Australia; see peptide sequences in Fig. S1). Plates were coated with each individual peptide at 20ug/ml in PBS overnight at 4°C. After blocking with PBS 1%BSA, purified anti-PF4 antibodies or human recombinant anti-PF4 antibodies (15 µg/ml) were added to the plates. The remaining steps were described above.

Recombinant antibody production

Anti-PF4 recombinant antibodies were generated by GenScript (Piscataway, NJ08854).[6] DNA fragments encoding full length IgG antibody heavy chain and light chain were synthesized and cloned into pcDNA3.4 vector separately, in frame with an artificial signal peptide in front. Resulted constructs were co-transfected into CHO cell at a HC:LC ratio of 1:1, and cultivated at appropriate condition. The culture medium supernatant was harvested by ultrafiltration, then subject to Protein A affinity purification and SEC-HPLC chromatography polishing. Antibody purity was characterized by both SDS-PAGE (reducing and non-reducing condition) and SEC-HPLC (TSKgel G3000SWxl column, mobile phase 0.1mol/L Na2SO4 in 0.1mol/L Phosphate Buffer, pH 6.7± 0.3). The purified antibodies were dialysis into 20 mM NaAC, 75 mM NaCl, 5 % sucrose, pH 5.5 and stored at -80 °C until required. A panel of recombinant antibodies with backmutations to germline (E31K) or change in IGLV haplotype were produced as described above.

Genome analysis of 100 patients with VITT

DNA extraction and whole genome sequencing

DNA was manually extracted from 100-200µl buffy coat (including PBMCs) using a HMW MagAttract Kit (Qiagen, Hilden, Germany). High molecular weight DNA was re-suspended in 80 -100 µl elution buffer AE (10 mM Tris-Cl ,0.5 mM EDTA pH 9.0). Input genomic DNA was quantified using a Quantifluor dsDNA kit (Promega, Fitchberg, USA) and ranged from 19 ng/µl to 83 ng/µl. Quality of genomic DNA was determined using a Genomic DNA Screen

Tape on an Agilent Tape Station 4200 (Agilent, Santa Clara, USA). DNA Integrity numbers (DIN) ranged from 6.8 to 9.8. As input for the TruSeq DNA PCR-free kit (Illumina, San Diego, USA) 1µg DNA was used according to the manufacturers reference guide #1000000039279 v00. Resulting libraries were inspected on the TapeStation 4200 with the D5000 Screen Tape (Agilent, Santa Clara, USA) and quantified using qPCR with a KAPA Library Quantification Kit (Roche, Basel, Switzerland). Libraries were sequenced on an Illumina NovaSeq 6000 S4 Flowcell using 150bp paired-end reads with a final loading concentration of 300 pM and 22 samples per Flowcell.

Whole genome sequence alignment, variant calling and haplotypes estimation

NGS data processing, variant calling and genotyping steps were performed in accordance to the best practice workflows recommendations of the Genome Analysis Toolkit (GATK)[7-9] using tool version 4.2.6.1. These steps included mapping of raw reads using BWA-MEM[10] to the hg38 reference genome (GRCh38 genome assembly from GATK resource bundle),[11] followed by marking read duplicates and the detection and correction for patterns of systematic errors in the base quality scores (recalibration with dbsnp138). Variant calling was initially performed for each sample individually using HaplotypeCaller in GVCF mode followed by consolidation across all samples and subsequent joint genotype calling for the whole sample set. Finally, variants were filtered by GATK's variant quality score recalibration with a sensitivity threshold of 99%. Additionally, Bcftools[12] was employed to determine allele frequencies at the sites of interest, which are likely to have somatic and not germline variants. To reconstruct the IGLV3-21 haplotypes of the patients with VITT we applied the PHASE software (v2.1)[13,14] ten independent times with seeds 2, 1,536, 2,936, 3,123, 4,957, 5,283, 6,757, 7,992, 8,633 and 9,045 for the random number generator to avoid seed-biased haplotype estimations.

Animal Experiments

Male and female triple-transgenic mice expressing human FcγRIIa and human PF4 with a murine PF4 knockout (B6(Cg);SJL-Pf4tm1 Tg(PF4) Tg(FCGR2A)) were passively immunized with recombinantly produced anti-PF4 IgG antibodies (1.5 µg/g bodyweight; anti-PF4 IgG group) or received either 0.9% NaCl or human control IgG (control group). Immunization was performed via retroorbital plexus for five consecutive days under short-term anaesthesia. Twelve hours after the first injection and then every twelve hours after the daily antibody injections 25µL EDTA blood for automated blood cell count analysis (VetScan HM5, zoetis Germany) was obtained. Experiments were performed according to local animal regulations (LALLFF MV 7221.3-1-009/24). Mice were euthanized during the experimental period when a humane endpoint was reached or at the planned endpoint 7 days after the last anti-PF4 IgG or control injection. Mice receiving the recombinant VITT antibody CR22046 died or reached a humane endpoint during the observation period. Numbers of mice measured at the corresponding time points are shown in Figure S7. For euthanasia, mice received an overdose of Ketamin/Xylazin and final bleeding was performed. Thrombosis was evaluated macroscopically during necropsy at a humane or the planned endpoint and microscopically in formaldehyde (Roti®Histofix, Carl Roth, Karlsruhe, Germany) 4 µm thin paraffin sections with HE-staining.

Supplementary Figures

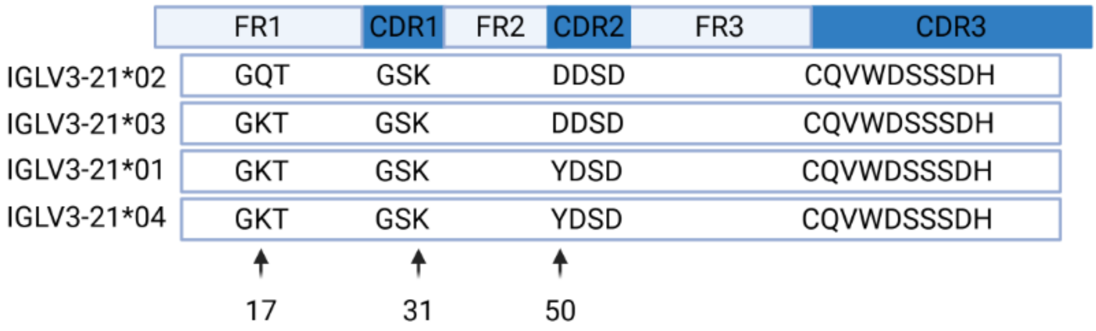

Figure S1. Alignment of IGLV3-21 light chain alleles.
Residue position according to the Kabat numbering.[15]

Pep1: MSILISPSNNTGWGL
Pep2: LISPSNNTGWGLRAP
Pep3: PSNNTGWGLRAPSKM
Pep4: NTGWGLRAPSKMYGG
Pep5: WGLRAPSKMYGGARQ
Pep6: RAPSKMYGGARQRST
Pep7: SKMYGGARQRSTQHP
Pep8: YGGARQRSTQHPVRV
Pep9: ARQRSTQHPVRVRGH
Pep10: RSTQHPVRVRGHFRA
Pep11: QHPVRVRGHFRAPWG
Pep12: VRVRGHFRAPWGALK
Pep13: RGHFRAPWGALKGRV
Pep14: FRAPWGALKGRVRSR
Pep15: PWGALKGRVRSRTTV
Pep16: ALKGRVRSRTTVDDV
Pep17: GRVRSRTTVDDVIDQ
Pep18: RSRTTVDDVIDQVVA
Pep19: TTVDDVIDQVVADAR
Pep20: DDVIDQVVADARNYT
Pep21: IDQVVADARNYTPAA
Pep22: VVADARNYTPAAAPV
Pep23: DARNYTPAAAPVSTV
Pep24: NYTPAAAPVSTVDAV
Pep25: PAAAPVSTVDAVIDS
Pep26: APVSTVDAVIDSVVA
Pep27: STVDAVIDSVVADAR
Pep28: DAVIDSVVADARRYA
Pep29: IDSVVADARRYARAK
Pep30: VVADARRYARAKSRR
Pep31: DARRYARAKSRRRRI
Pep32: RYARAKSRRRRIARR
Pep33: RAKSRRRRIARRHRS
Pep34: SRRRRIARRHRSTPA
Pep35: RRIARRHRSTPAMRA
Pep36: ARRHRSTPAMRAARA
Pep37: HRSTPAMRAARALLR
Pep38: TPAMRAARALLRRAR
Pep39: MRAARALLRRARRTG
Pep40: ARALLRRARRTGRRA
Pep41: LLRRARRTGRRAMLR
Pep42: RARRTGRRAMLRAAR
Pep43: RTGRRAMLRAARRAA
Pep44: RRAMLRAARRAASGA
Pep45: MLRAARRAASGASAG
Pep46: AARRAASGASAGRTR
Pep47: RAASGASAGRTRRRA
Pep48: SGASAGRTRRRAATA
Pep49: SAGRTRRRAATAAAA
Pep50: RTRRRAATAAAAAIA
Pep51: RRAATAAAAAIASMS
Pep52: ATAAAAAIASMSRPR
Pep53: AAAAIASMSRPRRGN
Pep54: AIASMSRPRRGNVYW
Pep55: SMSRPRRGNVYWVRD
Pep56: RPRRGNVYWVRDAAT
Pep57: RGNVYWVRDAATGVR
Pep58: VYWVRDAATGVRVPV
Pep59: VRDAATGVRVPVRTR
Pep60: AATGVRVPVRTRPPR
Pep61: ATGVRVPVRTRPPRT

Figure S2. Overlapping 15-mer peptides spanning the ChAdOx1 pVII protein.
A set of 15mer peptides with overlap of three residues for Chimpanzee adenovirus Y25 (ChAdOx1) pVII protein (Mimotopes, Melbourne, Australia). Immunoreactive peptide 32 is boxed.

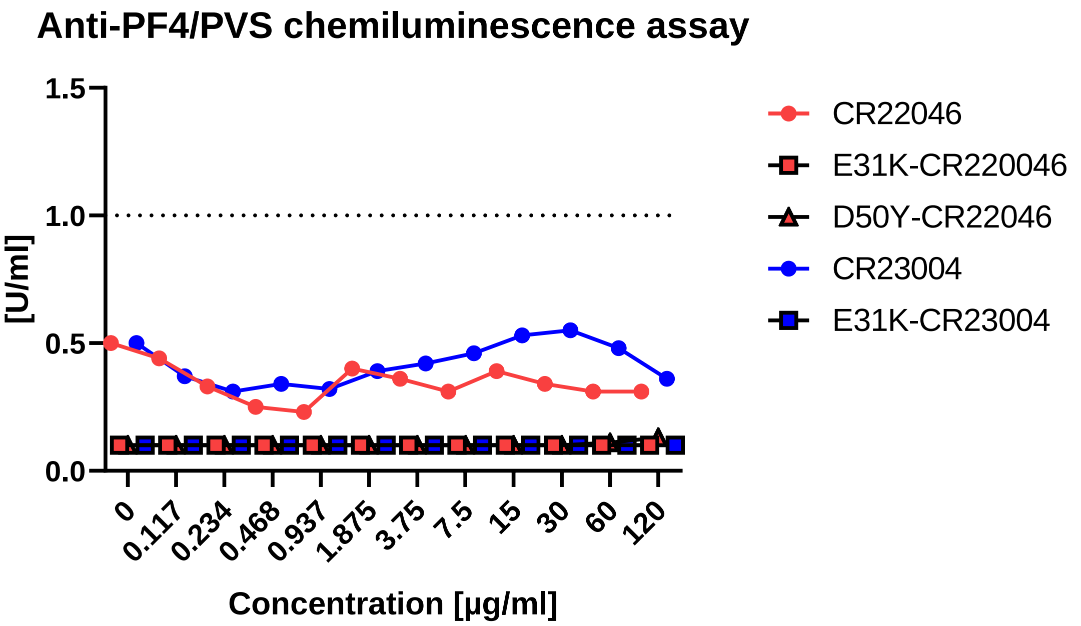

Figure S3. Anti-PF4/PVS chemiluminescence assay.

The recombinant anti-PF4 VITT antibodies CR22046 and CR23004 (red and blue circles); their E31K-variants E31K-CR22046 and E31K-CR23004, back-mutated to the germline sequence (red and blue squares); and the variant D50Y-CR22046, changed to an IGLV3-21*01/04 haplotype (red triangle) did not bind to PF4/polyvinylsulfonate (PVS) complexes at concentrations from 0.117-120 µg/mL in the HemosIL AcuStar HIT-IgG (AcuStar HIT-IgG$_{(PF4-H)}$ against PF4/polyvinyl sulfonate (PVS) complexes, Werfen, Barcelona, Spain). Dotted line shows the cutoff of the chemiluminescence assay. Antibody concentrations (x-axis) plotted as log^2-scale.

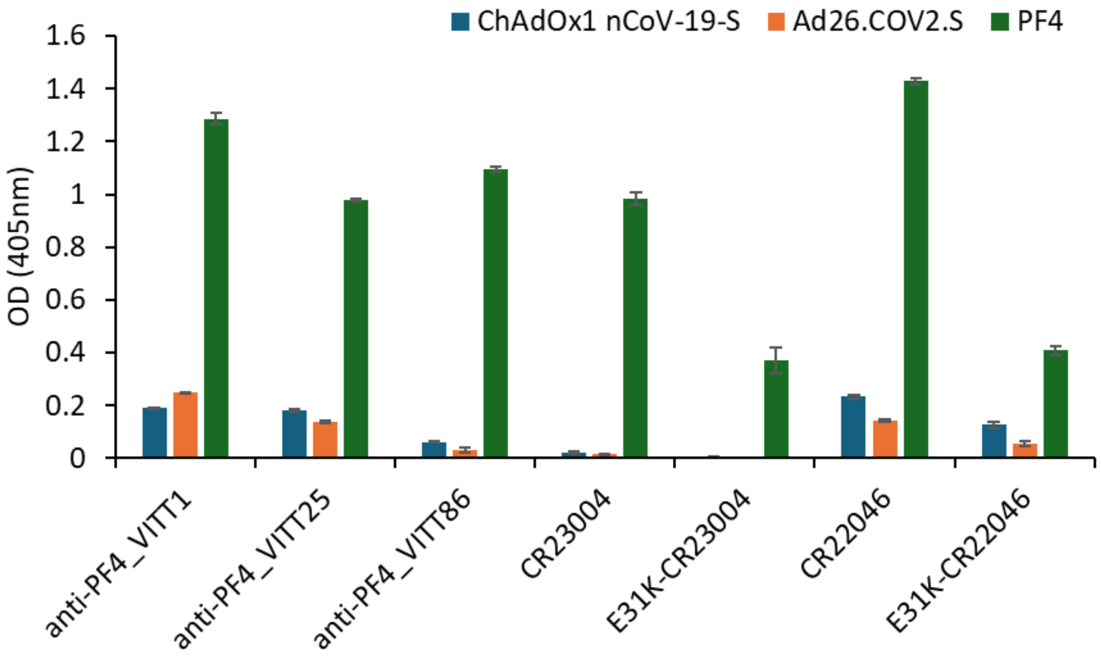

Figure S4. Anti-ChAdOx1 nCoV-19 virion, anti-Ad26.COV2.S virion, and anti-PF4 ELISA. The immunoreactivity of purified anti-PF4 antibodies and recombinant antibodies was assessed by ChAdOx1 nCoV-19-S virion, Ad26.COV2.S virion or PF4-coated ELISA.

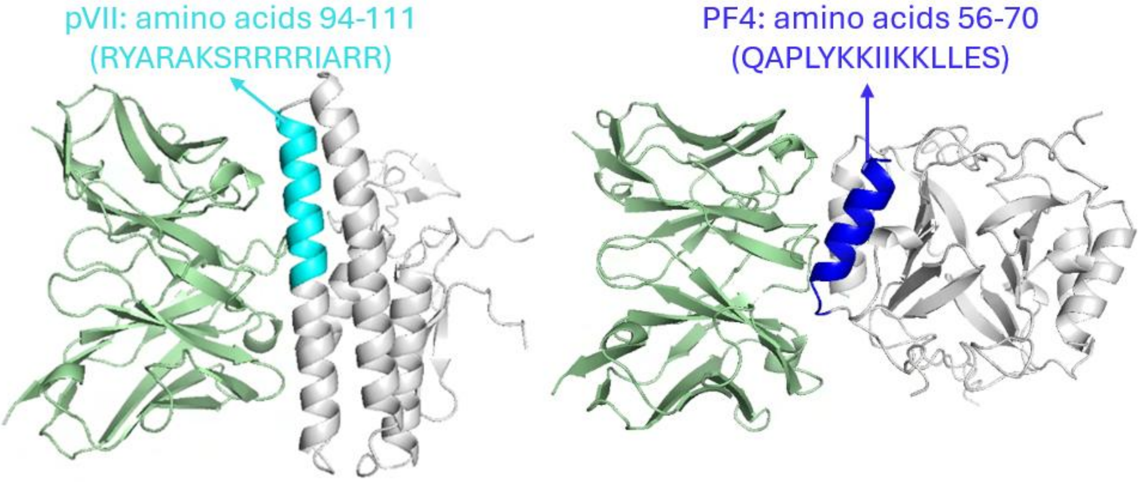

Figure S5. Structural mimics of pVII and PF4.
Structural mimics of pVII (in cyan) and PF4 (in blue) epitopes as short alpha helices is modeled by AlphaFold3 and visualized with PyMoL, consistent with mimicry through electrostatic similarity. Recombinant anti-PF4 antibody CR22046 (in pale green) is used as a representative antibody for the antibody-antigen modeling.

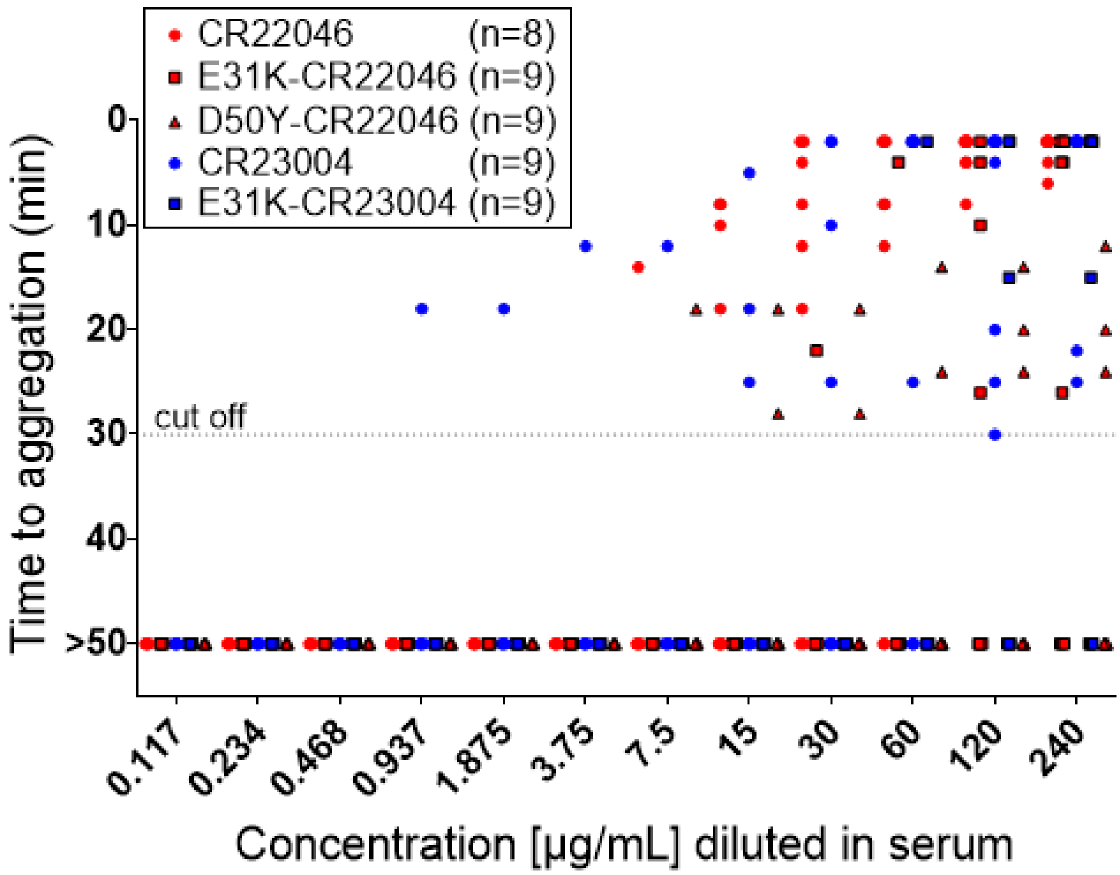

Figure S6. Functional platelet activation assay.
Individual data of the functional platelet activation assay (shown in Figure 1D) of recombinant anti-PF4 VITT antibodies CR22046 and CR23004 (red and blue circles); their E31K-variants E31K-CR22046 and E31K-CR23004, back-mutated to the germline sequence (red and blue squares); and the variant D50Y-CR22046, changed to an IGLV3-21*01/04 haplotype (red triangle). Antibody concentrations (x-axis) plotted as $\log^2$-scale. Data presented as individual points from platelets of eight or nine different donors. The dashed line shows the cutoff of the functional assay. Single data points of different platelet donors reacting with the same antibody might partially overlap.

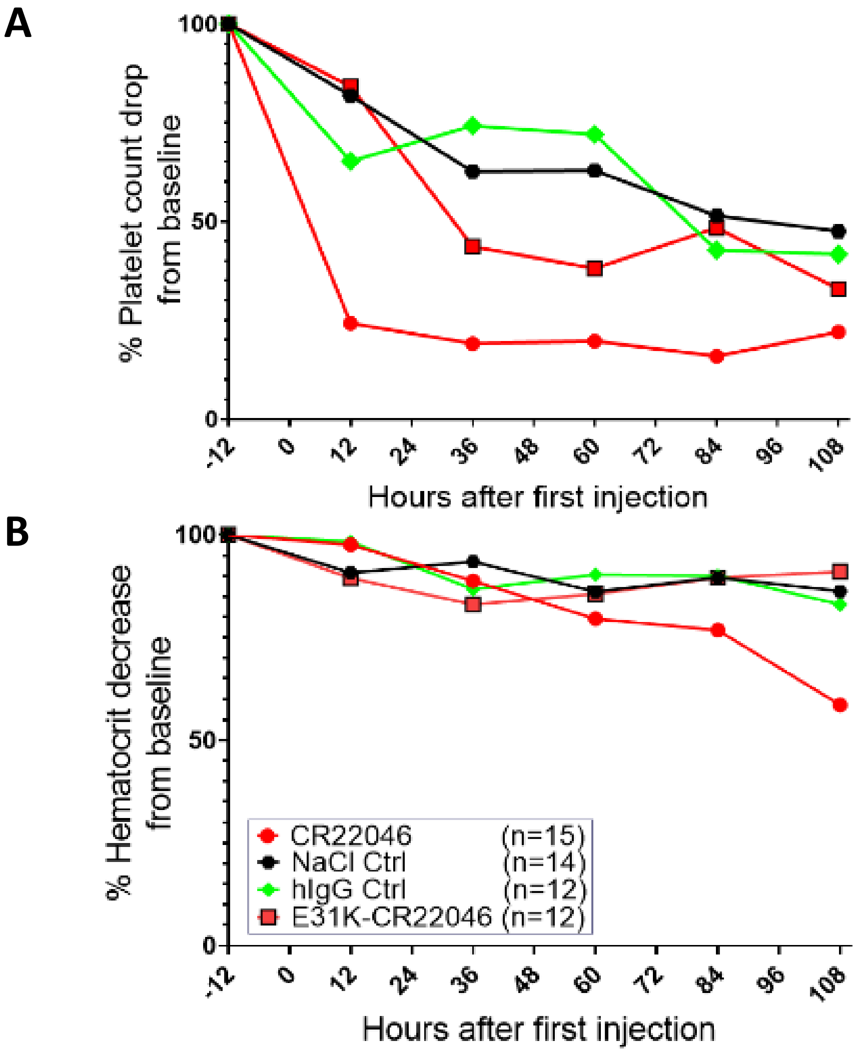

Timepoint	-12	12	36	60	84	108
CR22046	15	13	11	9	5	2
NaCl Ctrl	14	14	14	14	14	14
hIgG Ctrl	12	12	12	12	12	12
E31K-CR22046	12	12	12	12	12	11

Figure S7. Animal experiments.

A. Decrease of platelet counts (% from baseline) in mice transgenic for human PF4 and FcγIIa receptors, and knockout for mouse PF4 on a C57BL/6J background receiving CR22046 or E31K-CR22046 compared to human IgG (hIgG) Control or 0.9% NaCl as a second control group. CR22046 induced rapid and pronounced thrombocytopenia. The decrease in platelet counts in the control groups (hIgG and saline) most likely reflects an effect of daily blood draws. Data presented as mean from starting group size of 12-15 individual mice. Due to humane endpoints not all mice contributed to the later measurements, therefore the exact numbers of mice measured at the corresponding time points is added below B.

B. A decrease in hematocrit was most pronounced in the CR22046 group, likely caused by a higher frequency of major bleedings.

Data presented as mean from starting group size of 12-15 individual mice. Mice receiving the recombinant VITT antibody CR22046 died or reached a humane endpoint during the observation period. Numbers of mice measured at the corresponding time points are shown below the Figure.

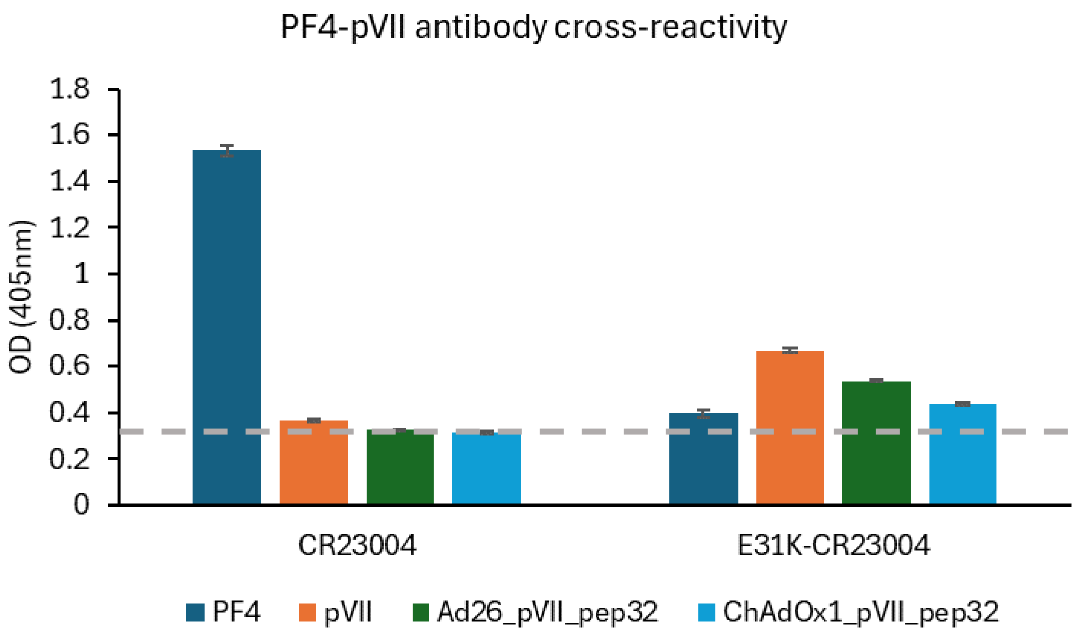

Figure S8: Binding of CR23004 and E31K-CR23004 to PF4, pVII and pVII peptides of Ad26 and ChAdOx1.

The recombinant VITT antibody CR23004 expressing the K31E mutation primarily bound to PF4; its counterpart, back-mutated to the IGLV3-21*02 germline sequence (E31K-CR23004) of the respective patient with VITT who received Ad26.COV2.S, lost PF4 binding and showed increased binding to ChAdOx1 pVII and the two pVII peptides Ad26-pVII **D**YAR**R**KSRRRRIARR and ChAdOx1-pVII **R**YAR**A**KSRRRRIARR. The bold letters highlight the amino acids in which the two peptides differ. Due to the experimental nature of the ELISA, we cannot provide a definite cutoff and show the binding of CR23004 to the Ad26-pVII peptide 32 and the ChAdOx1-pVII peptide 32 as dashed reference line.

Supplementary References

1. Schönborn L, Esteban O, Wesche J, et al. Anti-PF4 immunothrombosis without proximate heparin or adenovirus vector vaccine exposure. Blood 2023;142(26):2305-2314. DOI: 10.1182/blood.2023022136.
2. Krauel K, Preusse P, Warkentin TE, et al. Fibronectin modulates formation of PF4/heparin complexes and is a potential factor for reducing risk of developing HIT. Blood 2019;133(9):978-989. DOI: 10.1182/blood-2018-05-850370.
3. Wang JJ, Armour B, Chataway T, et al. Vaccine-induced immune thrombotic thrombocytopenia is mediated by a stereotyped clonotypic antibody. Blood 2022;140(15):1738-1742. DOI: 10.1182/blood.2022016474.
4. Wang JJ, Schönborn L, Warkentin TE, et al. Antibody Fingerprints Linking Adenoviral Anti-PF4 Disorders. N Engl J Med 2024;390(19):1827-1829. DOI: 10.1056/NEJMc2402592.
5. Wang JJ, Colella AD, Beroukas D, Chataway TK, Gordon TP. Precipitating anti-dsDNA peptide repertoires in lupus. Clin Exp Immunol 2018;194(3):273-282. (In eng). DOI: 10.1111/cei.13197.
6. Wang JJ, van der Neut Kolfschoten M, Rutten L, et al. Characterization of reverse-engineered anti-PF4 stereotypic antibodies derived from serum of patients with VITT. Blood 2024;143(4):370-374. DOI: 10.1182/blood.2023021307.
7. DePristo MA, Banks E, Poplin R, et al. A framework for variation discovery and genotyping using next-generation DNA sequencing data. Nat Genet 2011;43(5):491-8. DOI: 10.1038/ng.806.
8. Broad Institute. Genome Analysis Toolkit. (https://gatk.broadinstitute.org).
9. Van der Auwera GA, O'Connor BD. Genomics in the Cloud: O'Reilly Media, Inc., 2020.
10. Li H, Durbin R. Fast and accurate short read alignment with Burrows–Wheeler transform. Bioinformatics 2009;25(14):1754-1760. DOI: 10.1093/bioinformatics/btp324.
11. Broad Institute. Resource Bundle -Genome Analysis Toolkit. (https://gatk.broadinstitute.org/hc/en-us/articles/360035890811-Resource-bundle).
12. Danecek P, Bonfield JK, Liddle J, et al. Twelve years of SAMtools and BCFtools. GigaScience 2021;10(2). DOI: 10.1093/gigascience/giab008.
13. Stephens M, Smith NJ, Donnelly P. A New Statistical Method for Haplotype Reconstruction from Population Data. Am J Hum Genet 2001;68(4):978-989. DOI: 10.1086/319501.
14. Stephens M, Scheet P. Accounting for Decay of Linkage Disequilibrium in Haplotype Inference and Missing-Data Imputation. Am J Hum Genet 2005;76(3):449-462. DOI: 10.1086/428594.
15. Johnson G, Wu TT. Kabat database and its applications: 30 years after the first variability plot. Nucleic Acids Res 2000;28(1):214-8. (In eng). DOI: 10.1093/nar/28.1.214.
